# Evaluation of Nasolabial Aesthetics and Self-Image Satisfaction among 16- to 20-Year-Old Patients with Cleft Lip and Palate in Northeast Thailand

**DOI:** 10.1055/s-0044-1792018

**Published:** 2024-12-24

**Authors:** Palakorn Surakunprapha, Suteera Pradubwong, Kamonwan Jenwitheesuk, Poonsak Pisek, Bowornsilp Chowchuen

**Affiliations:** 1 Division of Plastic Surgery, Department of Surgery, Faculty of Medicine, Khon Kaen University, Khon Kaen, Thailand; 2 The Research Center of Cleft Lip-Palate and Craniofacial Deformities, Khon Kaen University, Khon Kaen, Thailand; 3 Divisions of Orthodontics, Department of Preventive Dentistry, Faculty of Dentistry, Khon Kaen University, Khon Kaen, Thailand

**Keywords:** evaluation, nasolabial appearance, cleft lip and palate, Thailand

## Abstract

**Background** Cleft lip and palate (CLP) impact nasolabial appearance, influencing the physical, psychological, and quality of life (QoL) of affected individuals. Evaluations of the nasolabial aesthetics by patients and medical professionals (both experienced and inexperienced) are critical for enhancing patient care.

**Methods** This cross-sectional study enrolled 32 patients aged 16 to 20 years with CLP who underwent continuous treatment at a university hospital in Thailand. Participants were asked to complete the Thaicleft QoL questionnaire for nasolabial aesthetic self-assessment and had their two-dimensional facial images captured and then evaluated by two groups of medical evaluators: four experienced and four inexperienced professionals. Data are presented as means and percentages, with analysis including standard deviations, Cronbach's α for evaluator consistency, kappa for interrater reliability, and the Wilcoxon signed-rank test to compare aesthetic judgments between the experienced and inexperienced medical evaluators.

**Results** Among the 32 patients, 19 (59.37%) were females, and 22 (68.75%) had unilateral CLP and 10 (31.25%) had bilateral CLP, all reporting high nasolabial aesthetic satisfaction. Inexperienced evaluators assigned higher aesthetic scores than their experienced counterparts ( *p* = 0.01), with statistically significant agreement among inexperienced evaluators in their assessments ( *p* < 0.05). Both group of evaluators demonstrated high reliability in terms of the lip.

**Conclusion** Experienced evaluators assigned lower aesthetic scores than inexperienced evaluators did. The patients themselves expressed high levels of satisfaction with their nasolabial appearance, indicating that the treatment outcomes were favorable from the patients' perspective.

## Introduction

Patients with cleft lip and palate (CLP) present with separation in the upper lip that may, in some cases, extend to the nasal or anterior palate. This condition results in aesthetic differences from the conventional facial appearance, significantly affecting the physical, psychological, and social quality of life (QoL) of those affected. 1

The incidence of CLP varies by ethnicity, with the highest rates observed in Asian populations, where it ranges from 0.82 to 4.04 cases per 1,000 newborns. In contrast, the incidence is 0.9 to 2.69 cases per 1,000 newborns in White populations and 0.18 to 1.67 cases in Black populations. 1 Specifically, in Thailand, the prevalence is documented at 2.14 cases per 1,000 newborns, with a slightly higher prevalence of 2.28 cases in the northeastern region of the country. 2

Surgical correction is as an important intervention in the management of individuals with CLP. 3 Patients typically undergo comprehensive treatment from infancy through adolescence, necessitating a holistic approach coordinated by a specialized interdisciplinary team within a cleft center, ensuring accessible treatment and meticulous patient record tracking, including care, surgical interventions, and rehabilitation, along with monitoring of treatment quality and continuity.

Cheiloplasty and rhinoplasty are essential for enhancing the facial aesthetics in patients with CLP. 3 Given its prominence, the nose, as the central feature of the face, plays a crucial role in facial aesthetics, which are not solely dependent on the lip but on the harmonious positioning of the nose and mouth. Adolescents with CLP often face social adjustment challenges because of their facial appearance. 4 During the ages of 8 to 17 years, a critical period for orthodontic intervention, these individuals exhibit resilience in coping with anxiety, depression, and self-awareness, yet continue to confront issues related to speech and facial aesthetics. 5 6 Among 33 patients with an average age of 17 years, concerns varied with the severity of the cleft but commonly included lip aesthetics, nose appearance, and speech. 7 Patients aged 11 to 18 years experienced bullying due to their appearance and expressed dissatisfaction with their facial appearance postsurgery. 8 The interplay between aesthetics and QoL was deemed crucial to the social integration of patients aged 14 to 18 years, with individuals aged 14 to 25 years exhibiting moderate satisfaction with their facial and dental aesthetics and the lowest satisfaction with their nasal appearance. 9 10

Satisfaction levels were noted to be higher among male patients than among female patients. 10 Orthodontic interventions have been recognized to enhance the QoL of patients, with treatment options including growth modification, orthognathic surgery combined with orthodontic treatment, and camouflage orthodontic treatment, selected based on the severity of malocclusion. Orthodontists are tasked with devising a treatment plan that optimizes aesthetics, functionality, and stability. 11 The current study included a sample of patients with unilateral or bilateral CLP who underwent orthodontic treatment, with some patients receiving orthognathic surgery.

Numerous studies have correlated aesthetics with QoL revealing a tendency toward lower aesthetic evaluations in this patient group. These findings underscore the necessity for comprehensive evaluations using a patient-reported outcomes approach. 8 9 10 Research involving children aged 8 to 15 years with CLP indicated a diminished QoL compared with their peers, 12 while adults aged 18 to 30 years reported lower-than-expected aesthetic satisfaction. 3 An investigation into the QoL and nasolabial appearance of 10-year-old patients used Thaicleft QoL questionnaires, 13 and the findings showed that patient families' satisfaction levels were on par with those of five experts assessing nasolabial appearance (scores ranging from 1 to 2.5, indicative of favorable aesthetics). 14

Assessing patients aged 16 to 20 years undergoing orthodontic treatment is instrumental in formulating personalized treatment plans, including surgical interventions. Evaluations by both experienced and inexperienced medical professionals offer additional insights. They expressed their different perceptions of the surgical outcome. These insights lead to improvements in patient treatment, which is crucial for the medical team to be aware of. This ensures that patients are satisfied with their appearance during subsequent surgeries and enhances their QoL. Therefore, this study aimed to determine the outcome of aesthetic appearance to improve treatment care in the future.

## Methods

This cross-sectional study was conducted from November 2020 to December 2021 at the Tawanchai Cleft Center, Center of Excellence in Cleft Lip and Palate at Khon Kaen University, Thailand, and enrolled patients aged 16 to 20 years who had undergone primary surgery for CLP and were receiving continuous follow-up and orthodontic treatment.

The eligibility criteria included patients with unilateral or bilateral CLP who received primary surgery, bone grafting, and, in some cases, cleft lip and nose correction at the center. These patients had no comorbidities and consented to participate in this study. For individuals under the age of 18 years, guardian permission was required for enrollment.

The sample size was determined based on the treatment outcome results from a previous study on QoL and nasolabial appearance in 10-year-old patients with CLP, 13 which had a standard deviation of 1.39 ( *Zα* /2 = 1.96, *σ* ^2^ = 1.39, *d* = 0.45, *N* = 231). Using the following formula, the calculated sample size was 32 patients:



Participants assessed their QoL using the Thaicleft QoL satisfaction questionnaire, a tool developed through a literature review in the QoL domain. This questionnaire comprises 24 items and has been validated with a reliability rating of 0.861, 14 making it appropriate for both the Thai population and populations in developing countries. In this study, only four psychosocial dimensions were considered: self-perception, facial aesthetics, speech, and hearing abilities. Each participant dedicated approximately 15 minutes to complete the questionnaire and to have two-dimensional images taken according to international standards at the Tawanchai Cleft Center.

The evaluators consisted of eight medical professionals, divided into experienced and inexperienced groups in the surgical treatment of CLP. The experienced group included two plastic surgeons, an oral maxillofacial surgeon, and an orthodontist, each with over 5 years of experience in treating and performing surgery in patients with CLP. The inexperienced group comprised two general surgeons and two dentists. All evaluators were informed of the image evaluation process and practiced on a sample case to familiarize themselves with the evaluation principles and concepts.

### Evaluation Parameters

**Fig. 1:**
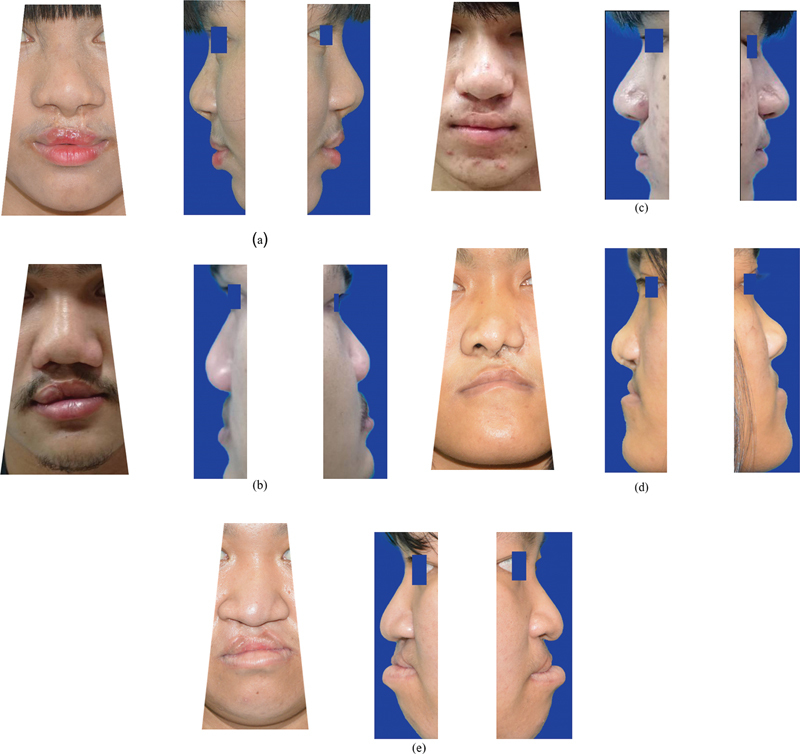
Examples of a patient's photographs for evaluation by experienced and inexperienced evaluators. ( **a** ) The example of a patient's photograph that received a good score (1–2.5 score) from the experienced evaluator. ( **b** ) The example of a patient's photograph that received a fair score (2.6–3.5 score) from the experienced evaluator. Note: No patient's photograph received a poor score (3.6–5 score) from the experienced evaluator. ( **c** ) The example of a patient's photograph that received a good score (1–2.5 score) from the inexperienced evaluator. ( **d** ) The example of a patient's photograph that received a fair score (2.6–3.5 score) from the inexperienced evaluator. ( **e** ) The example of a patient's photograph that received a poor score (3.6–5 score) from the inexperience evaluator.

The nasolabial appearance was evaluated using standard and internationally accepted two-dimensional images, emphasizing the lip, nose, and bilateral nasolabial regions ( Fig. 1 ). To ensure clarity of appearance details, three photographs were captured for each patient against a blue backdrop; these images were subsequently cropped to mitigate bias. 15 Aesthetic evaluation entailed separate assessments of the lips and nose, in addition to a comprehensive review of the nasolabial area. The evaluation criteria included the symmetry and fullness of the lips, integrity of the upper lip continuity, visibility of postsurgical scars, symmetry of the nasal tip, and appearance of the columella, alar regions, and nasal base, all in relation to the upper jaw. 16 Each image was assessed for 1 minute, followed by a 5-second break, until the entire set of 32 images was examined.

The aesthetic assessment by medical professionals was based on a scale established by Asher-McDade et al, 16 which ranged from 1 to 5; however, for this study, aesthetic ratings were categorized as follows: a score of 1 to 2.5 signified good aesthetics, 2.6 to 3.5 indicated fair aesthetics, and 3.6 to 5 indicated poor aesthetics. 15 Consensus among the evaluators was analyzed both within the groups and between the two groups concerning visual satisfaction with the lips, nose, and overall nasolabial region.

Patient-reported satisfaction with appearance utilized a 1 to 5 scale, where 1 represented the highest satisfaction and 5 denoted the lowest satisfaction. 13 In this study, the aggregate satisfaction ratings were interpreted as follows:

- Highest possible satisfaction: 1.51 to 2.50.
- High possible satisfaction: 2.51 to 3.50.
- Moderate satisfaction: 3.51 to 4.50.
- Low satisfaction: 4.51 to 5.00.

### Statistics Used for Analysis

Patient demographics and nasolabial appearance characteristics are expressed as percentages and means with standard deviations. The internal consistency among evaluators within each group was determined using Cronbach's α, whereas interrater reliability was assessed using the kappa statistic.

The Wilcoxon signed-rank test was used to evaluate differences in satisfaction regarding the aesthetics of the lips, nose, and overall nasolabial appearance within each group. Statistical significance was set at *p* < 0.05.

## Results

### Patient Demographics

The sample consisted of 32 patients and 59.37% ( *n* = 19) of the cohort were females. The mean age of the participants was 18.33 years. Treatment for unilateral CLP was administered to 68.75% ( *n* = 22) of the patients, and 81.25% ( *n* = 26) had undergone an alveolar bone grafting ( Table 1 ).

**Table 1:** General information of patients with cleft lip and palate (CLP) and their average age ( *n* = 32)

General information	Amount (%)
**Gender**	
Male Female	13 (40.63) 19 (59.37)
**Diagnosis**	
Unilateral CLP Bilateral CLP	22 (68.75) 10 (31.25)
**Alveolar bone graft (ABG)**	
Had received ABG Had not received ABG	26 (81.25) 6 (18.75)
**Orthognathic surgery (OGS)**	
Had received OGS Had not received OGS	3 (9.38) 29 (90.62)
**Occupation**	
Student Unemployed Other	25 (78.13) 3 (9.38) 4 (12.49)
**Guardian**	
Parents Grandparents and other	26 (81.25) 6 (18.75)
**Medical welfare/support**	
Universal Coverage Scheme/The Beautiful Smile Beautiful Voice Project Government	26 (81.25) 6 (18.75)
**Education**	
Elementary/high school Vocational certificate/high vocational certificate Bachelor's degree or higher Uneducated	2 (6.25)/ 17 (53.13) 5 (15.62)/- 4 (12.50) 4 (12.50)

Note: The average age was 18.33 years.

### Psychosocial Satisfaction

The cohort demonstrated the highest level of satisfaction with their facial appearance, as reflected in the satisfaction scores ( Table 2 ).

**Table 2:** Satisfaction score in the psychosocial aspect and its interpretation ( *n* = 32)

QoL in psychosocial aspect satisfaction	Mean ± SD	Interpretation
• Self-esteem • Self-image • Speech • Hearing abilities	1.81 ± 0.78 2.47 ± 0.62 2.31 ± 0.82 1.88 ± 0.79	Highest satisfaction level Highest satisfaction level Highest satisfaction level Highest satisfaction level
**Total**	2.18 ± 0.75	Highest satisfaction level

Experienced evaluators rated patients' facial aesthetics within the “fair” range for the lip, nose, and nasolabial region (2.73 ± 0.83, 2.60 ± 0.77, and 2.70 ± 0.86, respectively). The inexperienced evaluators rated these areas within the “good” range (2.53 ± 0.93, 2.13 ± 0.93, and 2.14 ± 0.78, respectively). The results were found to be statistically significant ( *p* = 0.01 and 0.03, respectively). Both groups of evaluators exhibited high agreement in lip assessment, as indicated by Cronbach's α coefficient greater than 0.70 ( Table 3 ).

**Table 3:** Cronbach's alpha test: verification of the degree of agreement among experienced and inexperienced evaluators for aesthetic evaluation of the lip, nose, and nasolabial region. ( *n* = 32)

Region	Number	Mean ± SD	Cronbach's α coefficient	*p* -value a
Lip (experienced)	1	2.90 ± 0.85	0.73	0.98
	2	2.90 ± 1.05		
	3	3.15 ± 0.88		
	4	1.96 ± 0.53		
Average score		2.73 ± 0.83		
Nose (experienced)	1	2.59 ± 0.87	0.71	0.68
	2	2.59 ± 0.75		
	3	3.21 ± 0.83		
	4	2.03 ± 0.63		
Average score		2.60 ± 0.77		
Nasolabial (experienced)	1	2.59 ± 0.91	0.52	0.39
	2	3.06 ± 1.16		
	3	3.25 ± 0.76		
	4	1.93 ± 0.61		
Average score		2.70 ± 0.86		
Average score (3 aspects)		2.67 ± 0.82		
Lip (inexperienced)	1	2.03 ± 0.64	0.71	0.06
	2	2.75 ± 0.76		
	3	2.56 ± 0.98		
	4	2.78 ± 0.60		
Average score		2.53 ± 0.93		
Nose (inexperienced)	1	1.87 ± 1.00	0.66	0.01*
	2	2.25 ± 0.74		
	3	2.12 ± 0.90		
	4	2.28 ± 0.92		
Average score		2.13 ± 0.93		
Nasolabial (inexperienced)	1	1.78 ± 0.75	0.62	0.03*
	2	2.31 ± 0.89		
	3	2.03 ± 0.82		
	4	2.46 ± 0.67		
Average score		2.14 ± 0.78		
Average score (3 aspects)		2.26 ± 0.88		

Abbreviation: SD, standard deviation.

a *p* -value means kappa statistic.

**Fig. 2:**
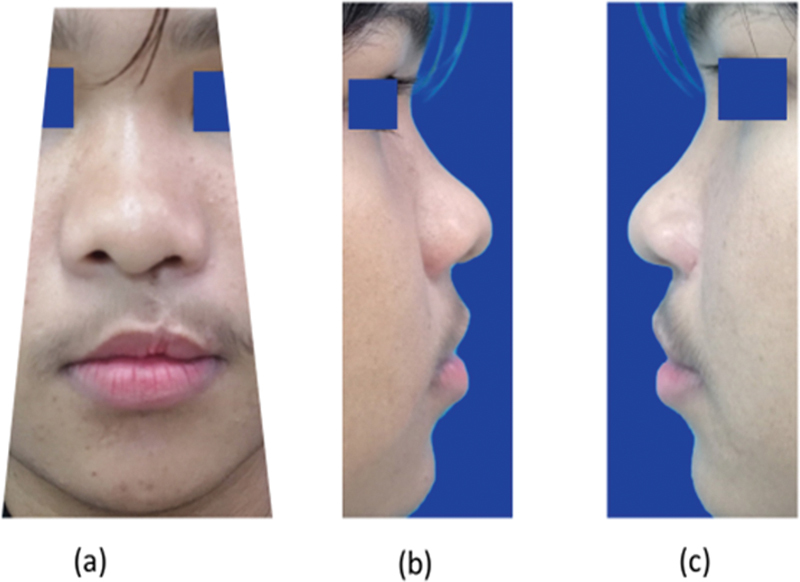
Representative standardized photographs taken of each patient. ( **a** ) Front view. ( **b** ) Left lateral view. ( **c** ) Right lateral view.

The aesthetic ratings for the lip provided by both experienced and inexperienced evaluators did not differ significantly. The ratings for the nose and overall nasolabial region, as assigned by the inexperienced evaluators, were statistically significantly higher than those given by their experienced counterparts ( *p* < 0.01; Fig. 2 ).

Ethical approval for this study was obtained from the Center for Ethics in Human Research, Khon Kaen University, Thailand (Ref. HE661407; Table 4 ).

**Table 4:** Wilcoxon signed-rank post hoc test: verification of the difference between experienced and inexperienced professional evaluators for the lip, nose, and nasolabial region ( *n* = 32)

Variable pairs	*n*	Mean ± SD	Minimum	Maximum	Interquartile range	*p* -value
Lip (experienced)	128	2.73 ± 0.95	1	5	2–3	0.19
Lip (inexperienced)	128	2.53 ± 0.81	1	4	2–3	
Nose (experienced)	128	2.60 ± 0.88	1	5	2–3	<0.01*
Nose (inexperienced)	128	2.13 ± 0.94	1	5	1–3	
NR (experienced)	128	2.71 ± 1.01	1	5	2–3	<0.01*
NR (inexperienced)	128	2.14 ± 0.82	1	4	2–3	

Abbreviation: NR, nasolabial region; SD, standard deviation.

* shows statistical significance of nose and NR evaluation by experienced and inexperienced evaluators.

## Discussion

This study involved a cohort of 32 patients, predominantly females, who primarily received treatment for unilateral CLP and frequently underwent an alveolar bone grafting as part of Thailand's Universal Coverage Healthcare Scheme. The majority of the participants were under parental care.

From a psychosocial perspective, in which patients assessed their overall appearance satisfaction, this cohort reported the highest satisfaction levels. Some studies revealed that patient-rated satisfaction within the 14- to 25-year age group expressed only moderate contentment with their facial aesthetics, 10 which is in line with findings from a study focusing on 10-year-old patients with CLP. 14 Interestingly, adolescents showed more concern for facial aesthetics than for speech functionality. 5 However, their speech must be addressed. 6 7 According to the patient's satisfaction outcome, the patients gave a high satisfaction score for nasolabial appearance, which is the same as that in the previous study. The findings showed that male patients had a higher satisfaction than female patients. 10

In the aesthetic evaluation conducted by both experienced and inexperienced evaluators, the results indicated that inexperienced evaluators assigned a high level to the lip, nose, and nasolabial region. Conversely, the experienced evaluators assigned fair level to these areas.

Aesthetic evaluations conducted during ongoing treatment do not necessarily provide a definitive assessment of the patient and nasolabial appearance. However, these evaluations are crucial for informing and optimizing future treatment plans for the patients. The perception of nasolabial aesthetics in patients treated for CLP is subjective and influenced by evaluators' perspectives. For instance, our more experienced medical professionals often gave lower aesthetic scores, possibly because of their comprehensive understanding of aesthetic principles and heightened expectations formed through extensive surgical and treatment experience.

While experienced evaluators showed high reliability in assessing lip and nose aesthetics, their consensus was lower for the nasolabial region. A study by Paiva et al 15 showed that experienced evaluators had a high level of agreement, with statistical significance ( *p* < 0.001). In our study, inexperienced evaluators also exhibited substantial agreement in their aesthetic judgments, with statistical significance ( *p* < 0.01). However, they show a low level of reliability. This aligns with the findings of Paiva et al, 15 in which the inexperienced evaluators' aesthetic satisfaction scores were markedly higher ( *p* < 0.001), suggesting that experienced evaluators might possess more stringent aesthetic standards due to their background in surgery and treatment.

The importance of assessing nasolabial appearance by both experienced and inexperienced evaluators offers various perspectives on nasolabial appearance. The outcomes of these evaluations allow experienced evaluators to view alternative viewpoints beyond their own expertise. Consequently, this can lead to advancements in surgical treatments, resulting in more favorable outcomes. This will benefit both patients and the medical teams in the future.

This study demonstrates the concept of self-image evaluations conducted by patients themselves including experienced and inexperienced evaluators. However, the outcomes of these groups were not directly comparable because of the use of different assessment tools. Our study also lacked a preoperative assessment of patient satisfaction, resulting in potentially unreliable outcomes. This limitation arises because some patients may have undergone surgical correction or received other treatments prior to the study, whereas others may have never received any surgical intervention. Consequently, the satisfaction levels of patients vary, with some patients exhibiting higher satisfaction with their treatment than others. Additionally, the Cronbach α coefficient for evaluations of the nose and nasolabial region was found to be below 0.70, indicating that the data may not be a reliable representation of the population. Future studies should focus exclusively on patients with either unilateral or bilateral CLP.

The cohort of patients in this study expressed high levels of satisfaction with their facial aesthetics. Regarding the evaluators, it was found that inexperienced evaluators assigned scores indicative of good aesthetics with a statistically significant reliability observed in the evaluation of the lips and the nasolabial region. Conversely, experienced evaluators assigned scores that were representative of fair aesthetics and exhibited high group reliability, although this did not reach statistical significance.

Aesthetic assessments of patients with CLP undergoing treatment are influenced by various factors, such as evaluator experience, parental perspectives, societal standards of nasolabial appearance, and the patients' personal views on beauty. Consequently, the concept of beauty requires further exploration and discussion from various perspectives.
